# Genetic stratification of depression in UK Biobank

**DOI:** 10.1038/s41398-020-0848-0

**Published:** 2020-05-24

**Authors:** David M. Howard, Lasse Folkersen, Jonathan R. I. Coleman, Mark J. Adams, Kylie Glanville, Thomas Werge, Saskia P. Hagenaars, Buhm Han, David Porteous, Archie Campbell, Toni-Kim Clarke, Gerome Breen, Patrick F. Sullivan, Naomi R. Wray, Cathryn M. Lewis, Andrew M. McIntosh

**Affiliations:** 1 Social, Genetic and Developmental Psychiatry Centre, Institute of Psychiatry, Psychology & Neuroscience, King’s College London, London, UK; 2 Division of Psychiatry, University of Edinburgh, Royal Edinburgh Hospital, Edinburgh, UK; 3 Lundbeck Foundation Initiative for Integrative Psychiatric Research, iPSYCH, Copenhagen, Denmark; 4 Institute of Biological Psychiatry, Mental Health Services Capital Region of Denmark, Copenhagen, Denmark; 5 NIHR Maudsley Biomedical Research Centre, South London and Maudsley NHS Trust, London, UK; 6 Department of Clinical Sciences, University of Copenhagen, Copenhagen, Denmark; 7 Lundbeck Foundation’s Center for GeoGenetics, GLOBE Institute, University of Copenhagen, Copenhagen, Denmark; 8 Department of Biomedical Sciences, Seoul National University College of Medicine, Seoul, Republic of Korea; 9 Centre for Genomic and Experimental Medicine, University of Edinburgh, Edinburgh, UK; 10 Centre for Cognitive Ageing and Cognitive Epidemiology, University of Edinburgh, Edinburgh, UK; 11 Usher Institute for Population Health Sciences and Informatics, University of Edinburgh, Edinburgh, UK; 12 Department of Medical Epidemiology and Biostatistics, Karolinska Institutet, Stockholm, Sweden; 13 Department of Genetics, University of North Carolina, Chapel Hill, NC USA; 14 Department of Psychiatry, University of North Carolina, Chapel Hill, NC USA; 15 Queensland Brain Institute, University of Queensland, Brisbane, Queensland Australia; 16 Department of Psychology, University of Edinburgh, Edinburgh, UK

**Keywords:** Depression, Genetics

## Abstract

Depression is a common and clinically heterogeneous mental health disorder that is frequently comorbid with other diseases and conditions. Stratification of depression may align sub-diagnoses more closely with their underling aetiology and provide more tractable targets for research and effective treatment. In the current study, we investigated whether genetic data could be used to identify subgroups within people with depression using the UK Biobank. Examination of cross-locus correlations were used to test for evidence of subgroups using genetic data from seven other complex traits and disorders that were genetically correlated with depression and had sufficient power (>0.6) for detection. We found no evidence for subgroups within depression for schizophrenia, bipolar disorder, attention deficit/hyperactivity disorder, autism spectrum disorder, anorexia nervosa, inflammatory bowel disease or obesity. This suggests that for these traits, genetic correlations with depression were driven by pleiotropic genetic variants carried by everyone rather than by a specific subgroup.

## Introduction

Depression is a common mental health disorder characterised by persistent feelings of sadness or a loss of interest in day-to-day activities lasting for at least a 2-week period. These feelings can be accompanied by tiredness, changes in appetite, changes in sleep patterns, reduced concentration, feelings of worthlessness or hopelessness and thoughts of self-harm or suicide. Zimmerman et al.^1^ found that there were 170 different symptom profiles amongst 1566 participants diagnosed with major depressive disorder from the Rhode Island MIDAS project. This variety of different symptom profiles suggest that depression is highly heterogeneous^2^. Depression is also comorbid with many diseases including cancer^3^, cardiovascular disease^4^ and other psychiatric illnesses^5^. Stratification of depression, to address heterogeneity and comorbidity, may aid in providing valuable aetiological insights and improve treatment efficacy.

Studies aimed at stratifying depression have examined differences between melancholic and atypical depression^6^, differences between the sexes and recurrence of the disorder^7^ and used data from other traits, such as neuroticism^8^ and social contact^9^ to stratify depression. Twin-based studies^10^ and genome-wide association studies^11,12^ have shown depression to be heritable and genetically correlated with a number of other traits and disorders. This shared genetic component could be due to pleiotropic variants shared across all individuals but could also be as a result of a subgroup for the other trait within depression cases. For example, there is a genetic correlation of 0.33 (standard error = 0.03) between depression and bipolar disorder^13^. If this genetic correlation was due to pleiotropy, then several of the bipolar disorder variants would be carried by most depression cases. However, if this correlation was due to a subgroup, then a greater proportion of the bipolar disorder variants would only be carried by individuals in this subgroup. A subgroup could arise where there is a causal association, a shared molecular pathway, a misclassification between the traits, or an ascertainment bias in the diagnosis of depression.

**Fig. 1:**
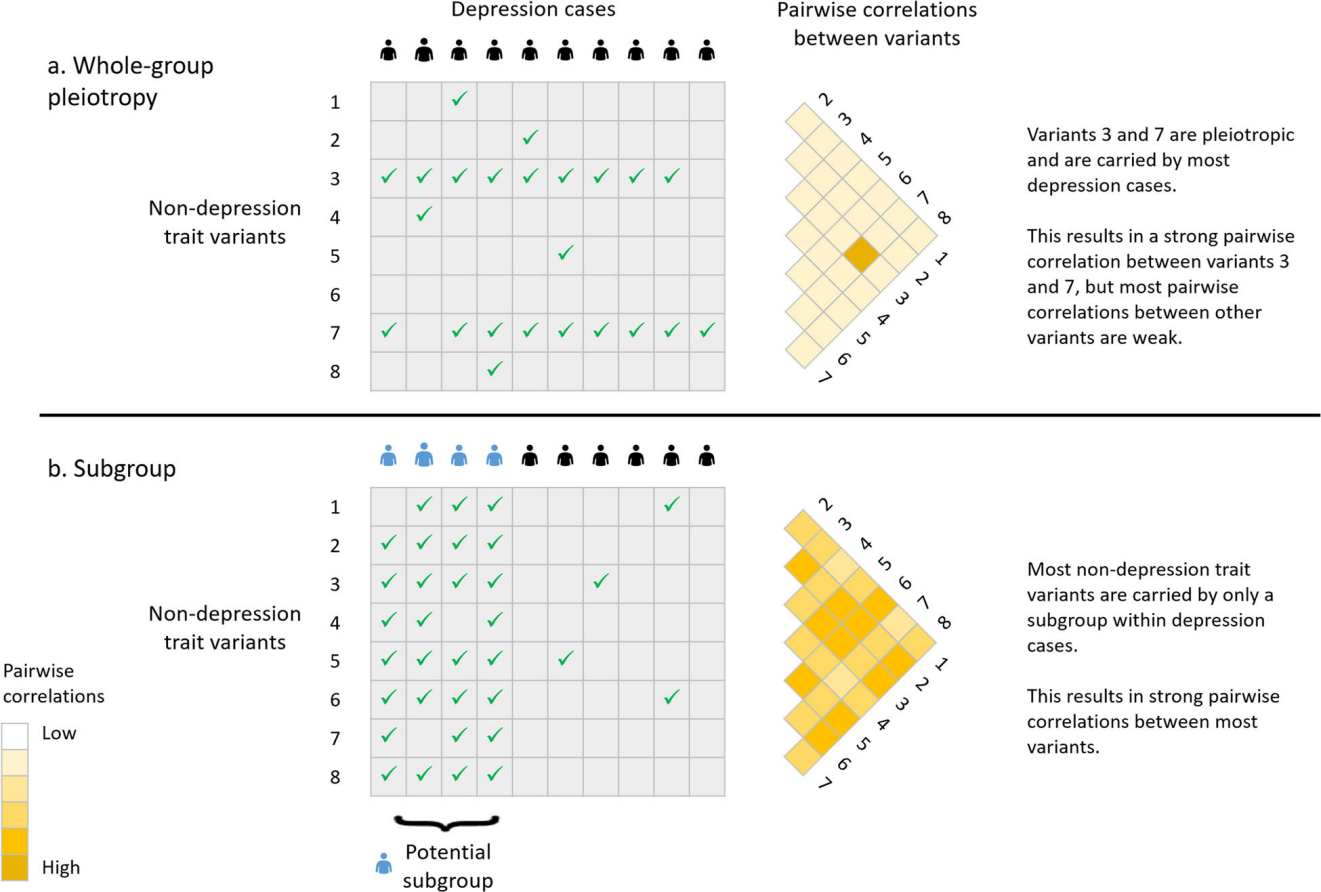
Comparison of the genetic architecture for whole-group pleiotropy and for a subgroup. Illustration of pairwise correlations between variants for **a** whole-group pleiotropy, where most depression cases carry a few variants associated with a non-depression trait and **b** a subgroup within depression cases (), where just the subgroup carry many of the non-depression trait variants. A tick indicates a depression case individual is a carrier of that non-depression variant.

For the current study, BUHMBOX (Breaking Up Heterogeneous Mixture Based On cross(X)-locus correlations)^14^ was used to determine whether there was evidence of a subgroup within depression that was genetically more similar to other traits. BUHMBOX uses variants associated with a subgroup trait to calculate weighted pairwise correlations of risk allele dosages within depression cases and controls, adjusted for effect size and allele frequency. Where there is a subgroup amongst depression cases that carry a greater proportion of the risk alleles for the non-depression trait, there will be consistent positive pairwise correlations between those variants (as illustrated in Fig. 1). BUHMBOX then calculates a *P*-value based on the likelihood of the observed pairwise correlations between variants.

Two definitions of depression were assessed in the UK Biobank^15^, one based on the Composite International Diagnostic Interview Short Form (CIDI-SF)^16^ and the other based on a broader help-seeking definition (broad depression)^12^. Since many traits are genetically correlated with depression^13^, a power calculation was performed to determine traits with sufficient power to detect a subgroup. Power is determined by the number of depression cases, the size of any subgroup within depression cases, the number of associated variants tested from the subgroup trait and the effect sizes of these variants. We tested sufficiently powered traits for evidence of a subgroup in depression cases using BUHMBOX v0.38^14^.

## Materials and methods

### UK Biobank cohort

The UK Biobank is a population-based cohort of 501,726 individuals with imputed genome-wide data for 93,095,623 autosomal genetic variants^15^. A genetically homogeneous sample of 462,065 individuals was identified using the first two principal components from a 4-means clustering approach. A total of 131,790 individuals were identified as being related up to the third degree (kinship coefficients >0.044) using the KING toolset^17^ and were removed from the sample. For these related individuals a genomic relationship matrix was calculated to enable the identification of one individual from each related group that could be reinstated. This allowed the reintroduction of 55,745 individuals providing an unrelated sample of 386,020 individuals.

### UK Biobank depression phenotypes

Two depression phenotypes were assessed for evidence of subgroups in UK Biobank. Both phenotypes were restricted to only those individuals that had completed the online mental health questionnaire (*n* = 109,049). The first phenotype analysed was based on the Composite International Diagnostic Interview Short Form (CIDI-SF)^18^ as used by Davis et al.^16^ to provide a lifetime instance measure of depression in the UK Biobank. Davis et al.^16^ provide a more in-depth description of this CIDI-SF phenotype, but in summary cases were defined as having:

- At least one core symptom of depression (persistent sadness (Data-Field: 20446) or a loss of interest (Data-Field: 20441)) for most or all days over a two-week period which were present “most of the day” or “all of the day”.
- Plus at least another four non-core depressive symptoms with some or a lot of impairment experienced during the worst 2-week period of depression or low mood.

The non-core depressive symptoms that were included in this assessment of the worst episode of depression were: Feelings of tiredness (Data-Field: 20449), Weight change (Data-Field: 20536), Did your sleep change? (Data-Field: 20532), Difficulty concentrating (Data-Field: 20435), Feelings of worthlessness (Data-Field: 20450) and Thoughts of death (Data-Field: 20437). Cases that self-reported another mood disorder were excluded. Controls were determined by not having at least one core symptom of depression or not endorsing at least another four non-core depressive symptoms if at least one core symptom was endorsed. This provided 25,721 CIDI-SF cases and 61,894 controls.

A second depression phenotype within the UK Biobank cohort was also examined using the broad depression definition from Howard et al.^12^ with detailed information provided in that paper. In summary, cases had sought help for nerves, anxiety, tension or depression from either a general practitioner or a psychiatrist (Data-Field: 2090 and Data-Field: 2100), whereas controls had not. Cases were supplemented with an additional 132 individuals identified as having a primary or secondary International Classification of Diseases (ICD)-10 diagnosis of a depressive mood disorder from linked hospital admission records (Data-Field: 41202 and Data-Field: 41204). Participants identified with bipolar disorder, schizophrenia or personality disorder and those reporting a prescription for an antipsychotic medication were removed. This provided a total of 36,790 broad depression cases and 70,304 controls. The phenotypic correlation between the CIDI-SF depression phenotype and the broad depression phenotype was 0.61 with the number of cases and controls shared across the two definitions shown in Supplementary Table 1.

### Sensitivity analysis

To allow a direct comparison between the two definitions of depression, the main analysis was restricted to those UK Biobank participants that had completed the mental health questionnaire. To examine whether the full UK Biobank sample provided greater power for the detection of subgroups, a sensitivity analysis was conducted using the broad depression phenotype (113,769 cases and 208,811 controls).

### Traits examined as subgroups within depression

We selected traits genetically correlated with depression (false discovery rate corrected, *q* < 0.01) in Howard et al.^13^ to test as subgroups within depression, which included anthropomorphic, autoimmune, life course, cardiovascular and other psychiatric traits. For each trait, there was a requirement that publicly available summary statistics were available and that the UK Biobank was not included in that study due to potential confounding effects (Supplementary Table 2).

The BUHMBOX power calculation test v0.1^14^ was used to determine whether there was sufficient power to detect a subgroup for each depression correlated trait and to identify the optimum variant selection criterion (*P* < 5 × 10^−8^, *P* < 10^−6^ or *P* < 10^−4^). The power calculation was conducted separately for the CIDI-SF depression phenotype and the broad depression phenotype. Variants from the summary statistics for each subgroup trait were examined in the UK Biobank. Variants that had a call rate <0.99, were out of Hardy–Weinberg equilibrium (*P* < 10^−10^), had a hard call threshold <0.25, or had a minor allele frequency <0.01 were excluded. BUHMBOX requires that all variants are available for all individuals and therefore individuals with a call rate <1 were removed. To identify independently segregating variants, clumping was conducted in PLINK v1.90b4^19^ using an *r*^2^ value of 0.01 across a 3-Mb window in either CIDI-SF or broad depression control individuals, respectively.

For the power analysis the approach used in Han et al.^14^ was followed, with 1000 simulated iterations run for each trait, the proportion of individuals in the subgroup was set to the genetic risk score beta coefficient (which represents the upper bound of the heterogeneity proportion) and a nominal subgroup *P*-value of 0.05 was used. Power analyses were used to identify the optimum variant selection criterion that provided the greatest power for each subgroup trait. Where power was the same across variant selection criteria, the strictest variant selection criterion was selected as the optimum. Variants with *P* < 10^−4^ were not publicly available for Squamous Cell Lung Cancer or Lung Cancer and so *P* < 10^−5^ was used instead. Only those traits that had a power >0.6 (using the optimum variant selection criterion) were selected to be tested for evidence of a subgroup within depression. A linear regression was used to examine the association between power and the heritability of each subgroup trait and the genetic correlation each subgroup trait shares with depression.

### Testing for subgroups within depression

For the traits that had power >0.6, variants meeting the optimum variant selection criterion were extracted from the UK Biobank. The same quality control thresholds and method to identify independently segregating variants as used as previously in the power analysis were applied. BUHMBOX v0.38^14^ was used to examine shared risk alleles for each subgroup trait within CIDI-SF depression and broad depression. BUHMBOX uses the positive correlations between risk allele dosages in cases to determine whether any sharing of risk alleles is driven by all individuals (whole-group pleiotropy) or by a subset of individuals (Fig. 1). The likelihood of observing such positive correlations are used to determine the subgroup *P*-values.

Sex, age, genotyping array and the first 20 principal components were fitted as covariates in the subgroup analysis. Bonferroni correction was used to account for the multiple testing of subgroup traits, with *P*-values <7.14 × 10^−3^ (0.05/7) or <0.01 (0.05/5) deemed significant for CIDI-SF or broad depression, respectively. No multiple testing correction was applied for the two depression definitions analysed. In the sensitivity analysis, using the full UK Biobank sample, a *P*-value <8.33 × 10^−3^ (0.05/6) was deemed significant for broad depression.

## Results

### Power analyses of potential subgroups traits

To determine whether there was sufficient power (>0.6) to detect a subgroup and identify the optimum variant selection criterion (*P* < 5 × 10^−8^, *P* < 10^−6^ or *P* < 10^−4^) for each trait the BUHMBOX power calculation test v0.1^14^ was used. The genetic risk score beta coefficients, representing an upper bound for heterogeneity proportion, for each trait within either Composite International Diagnostic Interview Short Form (CIDI-SF) depression or broad depression are provided in Supplementary Table 3. The results of the power analysis for detecting a subgroup for 25 available traits within the two depression definitions are provided in Table 1. Five traits had power >0.6 across both the CIDI-SF depression and broad depression definitions: bipolar disorder^20^, attention deficit/hyperactivity disorder^21^, autism spectrum disorder^22^, anorexia nervosa^23^ and inflammatory bowel disease^24^. There were two further traits, schizophrenia^25^ and obesity 3^26^, that had power >0.6 for detection of a subgroup in CIDI-SF depression.

**Table 1:** Power analysis for detecting a subgroup for 25 traits within either Composite International Diagnostic Interview Short Form (CIDI-SF) depression or broad depression in the UK Biobank.

Subgroup trait	PubMed ID	Optimum variant selection criterion	Power	Optimum variant selection criterion	Power
		CIDI-SF depression		Broad depression	
Neuroticism	24,828,478	<10^−4^	0.137	<10^−4^	0.120
Schizophrenia	25,056,061	<10^−6^	**0.607**	<10^−6^	0.306
Bipolar disorder	29,906,448	<10^−4^	**0.912**	<10^−4^	**0.727**
Attention deficit/hyperactivity disorder	30,478,444	<10^−4^	**0.912**	<10^−4^	**0.992**
Autism spectrum disorder	30,804,558	<10^−4^	**1**	<10^−4^	**1**
Anorexia nervosa	28,494,655	<10^−4^	**1**	<10^−4^	**1**
Triglyceride level	24,097,068	<10^−4^	0.183	<5 × 10^−8^	0.131
Coronary artery disease	26,343,387	<10^−4^	0.229	<5 × 10^−8^	0.071
Crohn’s disease	26,192,919	<10^−4^	0.193	<10^−4^	0.271
Inflammatory bowel disease	28,067,908	<10^−4^	**0.706**	<10^−6^	**0.665**
Waist to hip ratio	25,673,412	<10^−4^	0.070	<5 × 10^−8^	0.076
Body fat percentage	26,833,246	<10^−6^	0.057	<10^−6^	0.067
Waist circumference	25,673,412	<10^−4^	0.107	<10^−4^	0.070
Overweight	23,563,607	<10^−4^	0.131	<5 × 10^−8^	0.068
Obesity 1	23,563,607	<10^−4^	0.199	<10^−6^	0.089
Obesity 3	23,563,607	<10^−4^	**0.794**	<10^−4^	0.196
Body mass index	25,673,413	<10^−4^	0.101	<10^−4^	0.073
Age of menarche	25,231,870	<10^−4^	0.451	<5 × 10^−8^	0.081
Age of natural menopause	26,414,677	<10^−4^	0.407	<10^−4^	0.220
Years of schooling	25,201,988	<10^−4^	0.105	<10^−4^	0.089
College completion	25,201,988	<10^−4^	0.248	<10^−4^	0.160
Ever smoked	20,418,890	<10^−4^	0.081	<10^−4^	0.134
Age of smoking initiation	20,418,890	<10^−4^	0.061	<10^−4^	0.062
Squamous cell lung cancer^a^	28,604,730	<10^−5^	0.078	<5 × 10^−8^	0.085
Lung cancer^a^	28,604,730	<10^−5^	0.123	<10^−6^	0.137

PubMed identifiers (PubMed ID) for the 25 traits are provided. Bold values indicate that power was > 0.6. The optimum variant selection criterion that maximised power for the subgroup traits are provided.

^a^Variants with *P* < 10^−4^ were not publicly available for Squamous Cell Lung Cancer or Lung Cancer and so variants with *P* < 10^−5^ were tested instead.

A linear regression of subgroup power on the heritability of each subgroup trait and the genetic correlation shared with depression revealed that heritability was positively associated with power (CIDI-SF depression *P*-value = 5.32 × 10^−4^; broad depression *P*-value = 3.48 × 10^−4^), but genetic correlation with depression was not associated with power (CIDI-SF depression *P*-value = 0.57; broad depression *P*-value = 0.21).

The sensitivity analysis, analysing broad depression in the full UK Biobank sample, provided a small increase in power for the majority of subgroups compared to broad depression amongst individuals who had completed the mental health questionnaire. Six traits had power >0.6: bipolar disorder, attention deficit/hyperactivity disorder, autism spectrum disorder, anorexia nervosa, inflammatory bowel disease and schizophrenia (Supplementary Table 4).

### Testing for subgroups within depression

BUHMBOX v0.38^14^ was used to test seven traits for evidence of a subgroup within CIDI-SF depression, five traits within broad depression and six traits in the sensitivity analysis. The results of the subgroup for CIDI-SF and broad depression analyses are provided in Table 2 and the results of the sensitivity analysis are provided in Supplementary Table 5. None of the traits examined provided evidence of a genetic subgroup within depression (*P* > 0.05) before correction for multiple testing.

**Table 2:** Evidence of a subgroup from traits tested within either Composite International Diagnostic Interview Short Form (CIDI-SF) depression or broad depression in the UK Biobank.

Depression definition	Subgroup trait	Variants	*β*_GRS_	Depression cases/controls	Subgroup *P*-value
CIDI-SF	Schizophrenia	180	0.077	15,311/36,811	0.42
	Bipolar disorder	436	0.062	8140/19,466	0.62
	Attention deficit/hyperactivity disorder	342	0.028	8522/21,030	0.11
	Autism spectrum disorder	242	0.057	13,138/31,598	0.12
	Anorexia nervosa	169	0.016	16,024/38,388	0.47
	Inflammatory bowel disease	954	7.37 × 10^−3^	2186/5265	0.46
	Obesity 3	61	0.038	22,096/53,312	0.55
Broad	Bipolar disorder	435	0.041	11,531/22,186	0.60
	Attention deficit/hyperactivity disorder	342	0.034	12,345/23,844	0.07
	Autism spectrum disorder	242	0.051	18,802/36,000	0.15
	Anorexia nervosa	169	7.87 × 10^−3^	22,946/43,644	0.79
	Inflammatory bowel disease	219	8.02 × 10^−3^	22,738/43,355	0.64

The number of individuals classified as depression cases and depression controls is provided. The number of variants assessed and the genetic risk score beta coefficient (*β*_GRS_, representing the upper bound of the heterogeneity proportion) using the optimum variant selection criterion for that trait (as provided in Table 1).

## Discussion

Depression is a heterogeneous mental health disorder and is comorbid with many other diseases and illnesses. Over the last few years, valuable progress has been made in understanding the underlying genetic architecture of depression^11,13,27^. Furthermore, stratifying depression using genetic data remains a key goal within the psychiatric genetics community^28^ and should lead to improved classification of mental health conditions and more efficacious treatment for patients. Machine learning^29,30^ and polygenic risk score^6,31^ approaches offer possible methods for stratification in mental health. In the current study, we used BUHMBOX^14^ to identify whether traits that were genetically correlated with depression were correlated due to a subgroup, i.e. the correlation was driven by a subset of depressed individuals who had a greater genetic loading for the trait. Evidence of a subgroup within depression may provide future opportunities for stratifying the disease.

To allow a direct comparison between stricter and broader definitions of depression two phenotypes were examined. For the subgroups examined across both definitions (and using the same variant selection criteria), CIDI-SF depression had greater upper bounds for the heterogeneity proportion for bipolar disorder, autism spectrum disorder and anorexia nervosa, whereas broad depression had a greater upper bound for the heterogeneity proportion for attention deficit/hyperactivity disorder. The heterogeneity upper bound was assessed using genetic risk scores, which suggests that a stricter definition of depression shared a larger genetic component with bipolar disorder, autism spectrum disorder and anorexia nervosa and the broader definition shared a genetic component with attention deficit/hyperactivity disorder. This supports the observations of Cai et al.^32^ for bipolar disorder, autism spectrum disorder and attention deficit/hyperactivity disorder using genetic correlations (although they did not assess anorexia nervosa). As there were no significant subgroups found within depression, no firm conclusions can be drawn on the effectiveness of using stricter or broader definitions to stratify depression.

The lack of evidence for subgroups within depression for the seven traits examined with BUHMBOX, suggest that the previously reported genetic correlations^13^ were the result of pleiotropy, i.e. a genetic variant is associated with multiple phenotypes. Pleiotropy can result from either horizontal pleiotropy (where a variant has direct effects on multiple phenotypes) or vertical pleiotropy (where a variant has an effect on a phenotype, then this phenotype influences further traits downstream)^33^. To assess the presence of vertical pleiotropy a technique known as Mendelian randomisation^34^ can be used. This technique has been applied previously to depression and the traits examined with BUHMBOX, and no evidence of vertical pleiotropy was found^13^. This indicates that the genetic correlations between depression and the seven traits examined as subgroups are likely due to horizontal pleiotropy. Gaining a greater understanding of the biological mechanisms associated with pleiotropic variants could be informative for improving our comprehension and treatment for both depression and the correlated traits.

A sensitivity analysis was conducted to investigate whether additional power for detection of subgroups within broad depression could be obtained by analysing the full UK Biobank sample (*n* = 322,580) compared to the subsample that had completed the mental health questionnaire (*n* = 109,049). Decreased power was observed for some subgroup traits using the full sample which was due to lower heterogeneity proportions (based on the genetic risk score beta coefficient) and fewer genetic variants available for analysis (as all variants are required to be known and so fewer were available in the full sample). For most subgroup traits greater power was available using the full sample, however most were still underpowered to run the subgroup analysis. Schizophrenia was the only subgroup trait that sufficiently increased in power to exceed the >0.6 threshold, although no evidence of a subgroup was found. The average increase in power using the full sample compared to the mental health questionnaire subsample was only 0.06. However, larger genome-wide association studies of the currently underpowered traits could allow their re-examination as subgroups within depression in the future. The power to detect a subgroup for a trait was also influenced by the trait’s heritability, but not its genetic correlation with depression. Therefore, there is the potential to assess additional highly heritable traits where a feasible subgroup may exist within depression.

The limitations of the current study include selection bias, whereby particular individuals are more likely to participate in population-based cohorts or complete additional assessments, such as the online mental health questionnaire. Participants of the UK Biobank are healthier and from less deprived areas than the general population^35^ and those that completed the mental health questionnaire had a lower genetic predisposition to severe depression than those who did not^36^. UK Biobank participants that had either a self-reported or a hospital diagnosis of schizophrenia or bipolar disorder were excluded in the current analysis, which may limit the potential for identifying subgroups for these disorders. Most of the traits that are genetically correlated with depression were not included in the subgroup analysis due to a lack of power (≤0.6). As increasing large genome-wide association studies become available, a greater number of variants will meet the required selection criteria, allowing additional traits to be tested for evidence of a subgroup within depression.

Depression is both polygenic and heterogeneous and stratification of the disorder may lead to improvements in treatment outcomes. We examined 25 traits genetically correlated with depression using individuals that had completed the UK Biobank mental health questionnaire. There were seven traits sufficiently powered to be tested as subgroups within CIDI-SF depression and five traits tested as subgroups within broad depression, although none of these provided evidence for a genetic subgroup within depression. Alternative methodologies for stratification of depression could also be examined (i.e. polygenic risk scores and cluster analysis) along with consideration of other potential stratifiers (i.e. depression severity, depressive symptoms and antidepressant treatment response).

## Supplementary information

Supplementary Table 1

Supplementary Table 2

Supplementary Table 3

Supplementary Table 4

Supplementary Table 5

## Data Availability

The R code for BUHMBOX v0.38 and BUHMBOX power calculation test v0.1 are freely available and downloadable from http://software.broadinstitute.org/mpg/buhmbox/.
